# Tissue diagnosis using power-sharing multifocal Raman micro-spectroscopy and auto-fluorescence imaging

**DOI:** 10.1364/BOE.7.002993

**Published:** 2016-07-11

**Authors:** Faris Sinjab, Kenny Kong, Graham Gibson, Sandeep Varma, Hywel Williams, Miles Padgett, Ioan Notingher

**Affiliations:** 1 School of Physics and Astronomy, University Park, University of Nottingham, Nottingham, NG7 2RD, UK; 2 School of Physics and Astronomy, University of Glasgow, Kelvin Building, Glasgow G12 8QQ, UK; 3 Circle Nottingham Ltd NHS Treatment Centre, Lister Road, Nottingham NG7 2FT, UK; 4 Centre of Evidence-Based Dermatology, Nottingham University Hospital NHS Trust, QMC Campus, Derby Road, NG7 2UH, UK

**Keywords:** (170.0170) Medical optics and biotechnology, (300.6450) Spectroscopy, Raman

## Abstract

We describe a multifocal Raman micro-spectroscopy detection method based on a digital micromirror device, which allows for simultaneous “power-sharing” acquisition of Raman spectra from *ad hoc* sampling points. As the locations of the points can be rapidly updated in real-time via software control of a liquid-crystal spatial light modulator (LC-SLM), this technique is compatible with automated adaptive- and selective-sampling Raman spectroscopy techniques, the latter of which has previously been demonstrated for fast diagnosis of skin cancer tissue resections. We describe the performance of this instrument and show examples of multiplexed measurements on a range of test samples. Following this, we show the feasibility of reducing measurement time for power-shared multifocal Raman measurements combined with confocal auto-fluorescence imaging to provide guided diagnosis of tumours in human skin samples.

## 1. Introduction

Surgery is the mainstay of treatment for many cancers and the modality most likely to cure patients. The main goal of cancer surgery is to remove the entire tumour while trying to leave in place as much healthy tissue as possible. However, one of the most difficult challenges is the accurate detection of tumour margins during surgery. Currently there is a lack of reliable intra-operative techniques for imaging tumour margins or assessment of resections specimens, and failure to remove the tumour cells increases the risk of tumour recurrence and need for re-operation, emotional stress to patients and healthcare costs [1].

Raman micro-spectroscopy (RMS) is an optical technique based on inelastic scattering of light that can measure chemical differences between healthy tissue and tumours. Diagnosis models with simultaneous sensitivity and specificity higher than 90% have been demonstrated for many cancer types, including skin [2–4], breast [5–7], oesophagus [8], and lung [9,10]. Despite being able to measure subtle spectral differences between tissue structures, RMS mapping of large biological samples is slow (tens hours to days), making it unsuitable for imaging large tissue specimens typically resected in cancer surgery. Adaptive sampling techniques can be used for measuring large samples by initially choosing random sampling locations, and subsequently generating points iteratively using interpolation information between measured points [11]. Another selective-sampling approach is multimodal spectral imaging (MSI) based on integrated RMS with stratified sampling points generation by auto-fluorescence imaging (AF) [12]. Tissue AF imaging, which has high sensitivity, high speed and low specificity, was used as a first step to determine the key morphological features of the sample with high spatial resolution. This information was then used to automatically select and prioritise the sampling points for RMS. With this sampling strategy, the number of spectra required for diagnosis of tissue resections of ~1 cm^2^ was reduced to 800-3000, depending on the complexity of the tissue sample [12,13].

A common approach for reducing further the measurement time in RMS is to increase the power of the excitation laser. Although high power CW near-infrared lasers are available (e.g. >3W at 785nm), the excitation power is limited to the damage threshold of the order ~150mW per sampling point for ex-vivo tissue measurements. An alternative use of the laser power is to divide the main laser beam into several beams to allow multiple Raman spectra to be measured simultaneously (i.e. “multiplexed” or “multifocal” RMS, terms used interchangeably in this paper). If the power density for each beam is similar to the power density of the single-beam RMS, which depends only on the total power of the laser, this “power-sharing” multifocal RMS can in principle increase the sampling speed by a factor equal to the number of laser beams. Working towards this goal, line-scanning excitation has been demonstrated for speeding up RMS, and naturally complements the geometry of a spectrometer entrance slit [14,15]. Extensions of this power-sharing approach into two-dimensions have been demonstrated using a grid/array of multifocal excitation points generated using either a micro-lens array [16], diffractive optical element [17], or liquid-crystal spatial light modulator (LC-SLM) [18] For each of the different methods of excitation, a corresponding method for spatially filtering the light before dispersion inside the spectrometer is usually considered to maintain good spectral resolution and stray-light rejection. For the multi-focal grid/array excitation schemes, a matched grid of apertures can replace the single entrance slit, and either measures the spectra directly on the CCD simultaneously [16,17], or can be switched into pre-defined patterns, overlapping on the CCD and subsequently unmixed in postprocessing [18]. More flexible sampling locations, which are not restricted to grids of points, and can also be rapidly changed in software, have also been demonstrated using LC-SLMs [19,20]. Although no spatial filtering was used for the Raman-backscattered light, simultaneous measurements of multiple polymer microparticles and bacterial spores was reported by guided sampling (bright-field images were used for selection of sampling points).

Here, we build upon the ideas of AF-RMS multimodal spectral imaging [12] and simultaneous power-sharing measurements from arbitrary sampling locations [19,20] in order to increase the speed and diagnosis accuracy of tissue specimens resected during skin cancer surgery. In the first step, confocal AF imaging was used to generate sampling points for the multifocal RMS. While a LC-SLM was utilized for creating a power-shared excitation pattern for RMS (similar to [18–20]), the spectrometer slit was removed and a digital micro-mirror device (DMD) was added to behave as a software-reconfigurable reflective pseudo-“slit”, as demonstrated in [21] for fluorescence spectroscopy. While DMDs have been previously used in Raman micro-spectroscopy as spectral modulators replacing the CCD [22] or for spatially-offset Raman spectroscopy [23], here the DMD is used in a novel way as a software configurable multi-slit pattern. The DMD can be programmed with an arbitrary binary “slit/pinhole” pattern, which is matched to the laser excitation pattern, ensuring that high qulity Raman spectra can be obtained from all laser beams (spectral resolution determined by the DMD pattern). In this paper we describe the performance of this new instrument, and demonstrate the feasibility of power-sharing multifocal MSI for the diagnosis of basal cell carcinoma (BCC) tumours in skin surgical resections.

## 2. Methods

### 2.1 Instrumentation

**Fig. 1:**
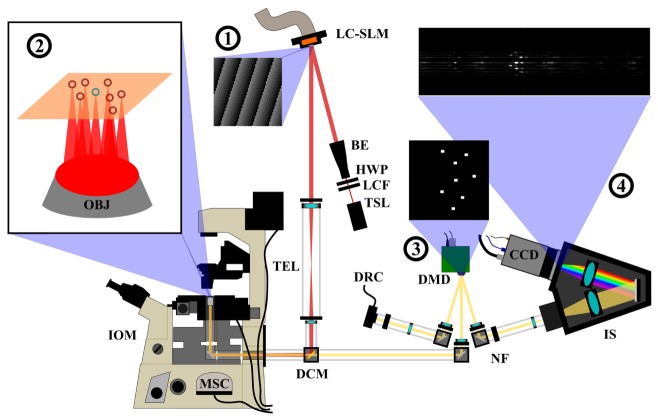
Schematic description of the multifocal RMS instrument. An LC-SLM phase hologram modulates the laser (1) creating an arbitrary excitation pattern in the sample plane shown in (2) (blue circle: 0th order beam on optical axis, red: 1st order reconfigurable off-axis beam). The sampling positions are synchronized with reflective slits on a DMD to spatially filter the Raman light (3) before dispersion onto a CCD inside the spectrometer (4). Glossary: Ti:sapphire laser (TSL), laser clean-up filter (LCF), half-wave plate (HWP), beam expander (BE), liquid-crystal spatial light modulator (LC-SLM), telescope (TEL), dichroic mirror (DCM), inverted optical microscope (IOM), microscope objective (OBJ), digital micro-mirror device (DMD), notch filter (NF) imaging spectrometer (IS), Raman CCD detector (CCD), microscope side-port camera (MSC), DMD inspection camera (DRC).

A schematic description of the multifocal RMS instrument is presented in Fig. 1

. A CW Ti:Sapphire laser (SpectraPhysics) with ~3W maximum output power at 785nm was expanded onto a 512 × 512 pixel LC-SLM (BNS XY phase series with 785nm dielectric coating, Boulder Nonlinear Systems USA). A phase hologram for producing the desired beam pattern was generated in LabVIEW, and displayed to the LC-SLM. The LabVIEW program for generating the phase holograms utilizes algorithms from the field of holographic optical tweezing (HOT), and involved a combination of grating and lenses algorithms, 2D Gerchberg-Saxton algorithm, amplitude modulation [24,25], calibrated LUT (specific to our LC-SLM), and the use of a blazing function for even distribution of power between sampling points (described in detail in [24]). Here however, as the maximum laser power is of importance for the Raman scattering intensity, the 0th order beam from the LC-SLM, which is usually excluded in HOT systems, was used as a fixed sampling point located close to the centre of the field of view, with the 1st order points being generated around it. This was to ensure that as much of the laser power available was utilized for multifocal measurements.

The modulated laser beam is then directed through a telescope towards the inverted microscope (Nikon Ti-S Eclipse equipped with a H107 ProScan II stage (Prior Scientific, UK) and Lumerera Infinity 2 camera), and through a 20 × /0.5 NA water-immersion objective (Leica, Germany), onto the sample. For Raman measurements, the samples were measured on either a MgF_2,_ CaF_2,_ or quartz coverslips (0.17 mm thick) mounted to a custom-built sample holder. The Raman back-scattered light was collected by the same objective and focused onto a DMD (Texas Instruments DLP Lightcrafter evaluation board, Farnell UK), modified to remove LEDs and projection optics (as described in [21]). A binary image displayed on the DMD display determines which direction the light is reflected at each pixel, either + 12° or −12° from normal. A rectangle is displayed to the DMD at the positions conjugated to each point on the sample selected for RMS measurements. This behaves as a reflective pseudo-slit, directing the Raman scattered photons from each sampling point towards the spectrometer. The size of the pseudo-slit can be adjusted by varying the dimensions of the rectangle displayed to the DMD to obtain the required spectral resolution. The rest of the field-of-view (FOV) is directed in the opposite direction, towards a CMOS camera used for calibration and inspection of the DMD (Thorlabs DCCM). To observe the sampling points, the DMD image values can be inverted, directing the sampling pattern towards the CMOS camera, which is used for calibration. During a measurement, the Raman-scattered beams are directed towards and then focused into a high-throughput imaging spectrometer (Acton LS785, Princeton Instruments USA) with a 1000 g/mm plane ruled diffraction grating (Richardson Gratings, USA), and dispersed onto a 256 × 1024 pixel CCD camera (Newton BR-DD, Andor UK). As the DMD now acts as the spatial filter for the spectrometer, the mechanical slit is opened to 2mm to allow the Raman light from the off-axis sampling points into the spectrometer. The Raman spectra are calibrated based on 5 peaks of a polystyrene sample measured by the 0th order beam in fingerprint region using a 3rd order polynomial fit.

The synchronisation of all components was controlled in LabVIEW, with an interactive calibration routine where the position of LC-SLM-generated laser spots was matched with points within the microscope camera FOV. The calibration of the DMD was then performed in a similar manner using the CMOS camera imaging the reflection of the DMD. The CCD vertical offset was also calibrated, such that the position of each dispersed sampling point was known relative to the fixed 0th order beam, and could be read out individually into a spectrum automatically.

For the MSI measurements, AF images (405 nm laser excitation, emission 450-480nm range) of tissue samples were acquired on a separate microscope (Nikon Ti-Eclipse) equipped with a C2 confocal fluorescence scanner. A transformation routine based on brightfield images recorded on both the multifocal RMS and confocal AF microscopes was developed to move between the stage co-ordinate systems of the two instruments. This transformation was necessary to generate the sampling locations in the correct co-ordinates on the multiplex instrument stage, instead of the AF microscope. An integrated instrument would eliminate the requirement for this transformation. The AF images were segmented using a thresholding method with maximum homogeneity for overall segments, and sampling points for RMS were generated based on an algorithm developed earlier [13]. These sampling points within the FOV (180 × 60 µm) were grouped into batches of 6 points using iterated *k*-means clustering. Additional checks on the co-ordinates were carried out to ensure no vertical overlap on the CCD between the dispersed Raman spectra (which would create cross-talk, typically separation of roughly 10 µm was sufficient). Due to the relatively small size of the FOV for our current instrument, many batches contained single points. For this reason, additional points were generated randomly within the FOV to ensure each batch had 6 sampling points. This is important as the current system uses the fixed full power of the beam, and thus reducing the number of simultaneous measurements would increase the laser power per sampling point, potentially above the damage threshold for the tissue. The factors limiting the FOV were the size of the DMD display, the maximum spectrometer slit width (2 mm), and the size of the optics in the collection path (25.4 mm diameter). The co-ordinates were were labelled by a number (from 1 to *N*) denoting their batch assignment as well as a binary identifier indicating which co-ordinate was assigned to be the 0th order (determined by measuring the co-ordinate closest to the batch centroid). This information was used in the automated LabVIEW software to move the stage to place the 0th order beam at the allocated location on the tissue, and the SLM would then automatically update to create the desired excitation pattern for the 1st order beams in that batch (with the corresponding DMD pseudo-slits). The CCD then acquired the spectra simultaneously during one acquisition window (1-2s integration time, 50 kHz read-out speed) using two horizontal tracks per sampling point, before moving to the next batch.

As there is not only a vertical offset of measured points on the CCD, but also an offset in the dispersion direction, the Raman shift axis was corrected for each measured spectrum. This was achieved by using the 320 cm^−1^ Raman peak corresponding to the MgF_2_ substrate. Due to this shift, the spectral range of the Raman measurements differed for each sampling point, and thus the spectral range was cropped to 520–1830 cm^−1^, ensuring the same number of data points in each spectrum.

### 2.2 Tissue samples

All skin tissue samples were obtained during Mohs micrographic surgery at the Nottingham University Hospitals National Health Service (NHS) Trust. Ethical approval was granted by the Nottingham Research Ethics Committee (07/H0408/172) and informed consent was obtained from all patients. The samples were kept frozen at −20°C until used for Raman spectral measurements. For each tissue sample, the diagnosis was based on adjacent haematoxylin and eosin (H&E) stained tissue sections. In total, samples from 15 patients with basal cell carcinoma on the face or neck were included in this study: 10 for the classification model and 5 for MSI.

### 2.3 Data postprocessing

Before any processing to the Raman spectral data, the recorded spectra were first calibrated to their relative wavenumbers, and the fingerprint region from 600 to 1800 cm^−1^ was selected [26]. Cosmic-rays were then removed from the spectra. All spectra were normalized to zero mean and unity of standard deviation.

In order to build a spectral data set for a diagnostic model, raster scanned Raman spectral images from the tissue blocks were used. These images, with sizes of 960 x 960 µm^2^ at 15 µm stepsizes, were clustered using *k*-means analysis, and compared to the adjacent H&E sections. The clustered regions were further processed to be well correlated to the H&E sections. For those spectra which are known to tissue diagnosis, labels were given with classes of BCC, epidermis, dermis and fat. and this process was repeated for tissue blocks from 10 patients.

The diagnostic model was built with a series steps; firstly, a quality control to remove the spectra when the intensities are saturated, as this is normally from the contamination and burnt to the samples; secondly, principal component analysis was used to identify the dye and fat spectra based on a threshold score corresponding to the second and third principal components; finally, a multinomial logistic regression classifier was used to discriminate BCC, epidermis, and dermis based on selected Raman spectral features. First, the areas under the following bands were calculated after subtraction of local linear baselines: A_1_ = 772 - 800 cm^−1^, A_2_ = 843 - 865 cm^−1^, A_3_ = 825 – 946 cm^−1^, A_4_ = 993 – 1022 cm^−1^, A_5_ = 1070 – 1115 cm^−1^, A_6_ = 1235 – 1279 cm^−1^ and A_7_ = 1279 – 1327 cm^−1^. These band areas were then used to calculate the ratios {A_1_/A_4_, A_2_/A_4_, A_3_/A_4_, A_5_/A_4_, A_6_/A_7_}, which were used as input for a multinomial logistic regression classifier. These spectral features were selected based on our previous study described in [4]. The regularization of the model was performed using 5-fold cross validation to justify the performance with target 95% sensitivity. The spectral measurements on multiple locations within a single segment were averaged into a single spectrum, which is then classified by the model.

## 3.Results

### 3.1 Performance of the DMD as a reflective pseudo-slit for Raman spectroscopy

**Fig. 2:**
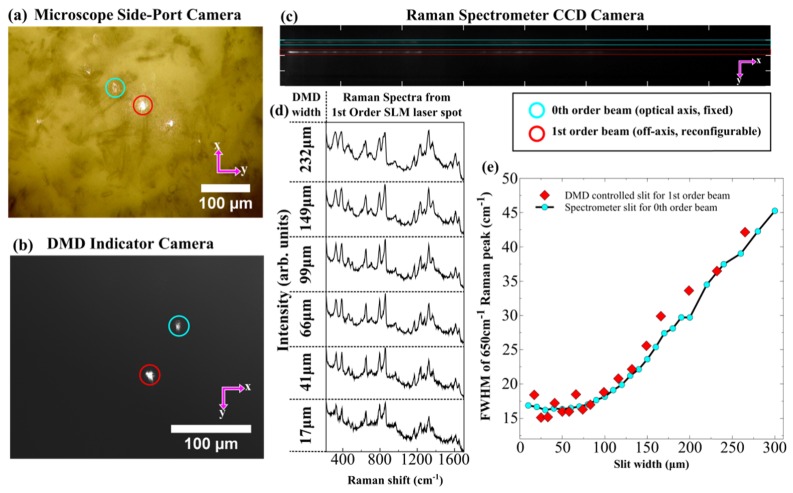
Testing the efficacy of a DMD as a pseudo-slit for Raman micro-spectroscopy: (a) Raman CCD image for a two-point system. (b) Image of the corresponding sample illumination, and (c) the DMD indicator camera (with mirrors inverted to direct only the sampling points towards the camera). Spectral resolution testing: (d) Raman spectra of a tylenol sample for different slit widths on the DMD for a first-order spot, and (e) comparison of the worsening of the spectral resolution for increasing slit width of the DMD-generated reflective slit, and traditional mechanical slit.

The application of a DMD as a reflective slit for Raman spectroscopy has not been demonstrated previously to the best of the authors knowledge. Thus the performance of the multiplexed RMS instrument in terms of spectral resolution should be tested and compared to a traditional mechanical (transmitting) slit. First, spectra of tylenol samples were acquired using no spatial light modulation (uniform hologram displayed to LC-SLM), in a single beam setup, with the DMD behaving as a simple mirror (i.e. all light directed towards the spectrometer). The mechanical slit was opened incrementally, and the full-width at half maximum of the 650 cm^−1^ Raman peak measured for each slit width (chosen due to the adequate isolation from other bands). Then, after opening the slit to 2mm, a single first-order LC-SLM beam was generated, and Raman spectra were acquired using various sizes of pseudo-slit on the DMD. Figure 2(e)

shows that the spectral resolution improves when the size of the slit is reduced, both for the DMD and mechanical slits. Furthermore, the spectral resolution achieved with the DMD matches the results obtained using the mechanical slit, indicating the suitability of the DMD as a reflective slit for multiplexed RMS.

A departure from the functionality of a traditional slit is that the DMD slit height can also be varied, but this does not affect spectral resolution (assuming the DMD is well-aligned with the dispersion axis of the spectrometer). The height was chosen to be roughly the same as the slit width, as increasing it only slightly improves the signal-to-noise for well-focused laser spots. However, this increase also limits the spacing on the CCD vertical axis, and hence the minimum distance between two sampling points. For samples with efficient Raman scattering, this height can be decreased, allowing an increased number of beams able to fit on the CCD. It should be noted that this DMD-based configuration also has the added advantage that any unwanted “ghost-order” LC-SLM spots and stray Raman scattered light can be rejected (towards the inspection camera).

### 3.2 Consistency of the Raman spectra within the field-of-view (FOV)

As the intensity of spontaneous Raman scattered light is proportional to the intensity of the excitation laser, it is desirable that the intensity of each beam in the multiplexed RMS should be similar, in order to obtain similar quality spectra. However, the power of each sampling point will decrease the further it is from the 0th order beam (i.e. the optical axis), due to the phase hologram requiring higher spatial frequencies for such beams, and losses due to off-axis imaging.The blazing function described in [24] can be used to some extent to distribute the power more evenly among the laser beams, as shown in section 3.4, mainly by reducing the power in the 0th order, distributing it into the higher order beams. The loss of Raman signal for the off-axis sampling points is also important to take into account, and can be reduced by decreasing the total optical path length and increasing the diameter of the optics used to image from the objective to the spectrometer.

**Fig. 3:**
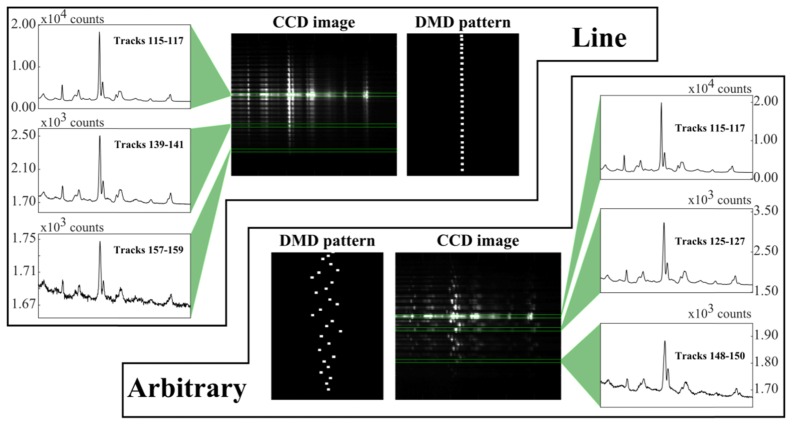
Homogeneity of multiplexed spectra on a uniform polystyrene substrate for line and arbitrary sampling patterns. CCD images (cropped, resized, and intensity-scaled for clarity) show Raman fingerprint region for line and arbitrary placement of spots, with selected spectra (the most intense corresponding to the 0th order/optic axis for each setup). Total laser power: 2.6W before LC-SLM; integration time: 1 second.

In order to test the variations in laser power, Raman spectra of a uniform polystyrene sample were measured by generating various patterns of laser beams within the FOV. The minimum separation between beams was restricted to 10µm in order to avoid overlap of spectra on the CCD. It should be noted that this is not a strict rule, and depends on the magnification of the optics used. The height of the slit for each beam was decreased to allow spectra to be obtained from only two vertical tracks on the CCD. Figure 3

shows the resulting CCD image and selected spectra. Analysis of the spectra by singular value decomposition did not detect any distortions of the Raman bands for spectra measured using the 1st order beams at different points within the FOV. Figure 3 shows that despite the use of a blazing function, the 0th order sampling point has ~10 × the signal of any 1st order beam. Furthermore, the variation in the signal from the 1st order spots, from those close to the optical axis (0th order) and the edge of the FOV, was another factor of ~10 × . This drop in signal was attributed to the typically lower power in the laser beams at the edge of the FOV as well as vignetting caused by the optical components in the collection path. However this variation in SNR is most apparent when using as many beams as possible, and the blazing function can produce a more even power distribution when a lower number of beams are used (see section 3.4).

### 3.3 Matching of sampling points to CCD tracks

**Fig. 4:**
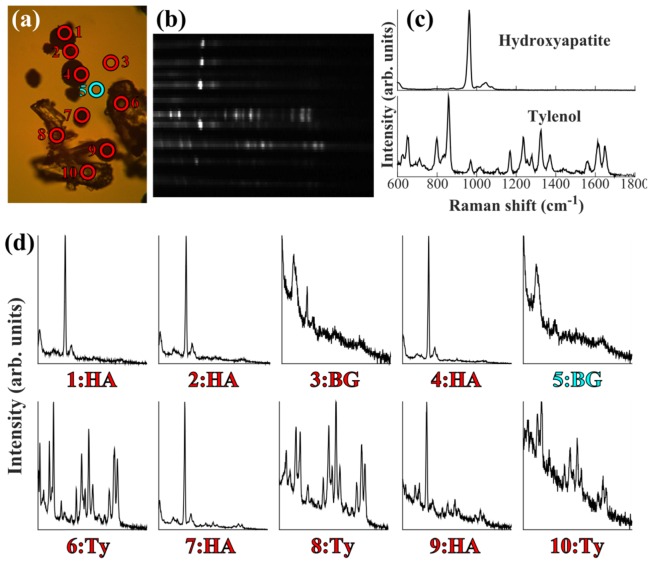
Multifocal Raman spectra of a mixed sample consisting of Tylenol and hydroxyapatite powder on a quartz coverslip (micrograph shown in (a)). Sampling points selected manually. Cropped CCD image of the multiplexed spectra (b), reference spectra (c) and the corresponding spectral plots from the labelled sampling points (BG: background, Ty: tylenol, HA: hydroxyapatite) (d). Laser power at sample: ~1W; integration time: 2 seconds.

To ensure that the Raman spectra recorded matched the selected positions on the sample, measurements were carried out on a heterogeneous mixture of powders. Figure 4

presents multiplex Raman spectra collected from a mixture of tylenol and hydroxyapatite (HA) powder deposited on an quartz coverslip.

The results in Fig. 4 show that the sampling points selected on the micrograph in (a) match with the Raman spectra shown in (d): points 1, 2, 4, 7 and 9 correspond to HA micro-particles, points 6, 8 and 10 correspond to Tylenol crystals, and the remaining points (3 and 5) are from the quartz substrate.

### 3.4 Raman spectra of animal tissue samples

**Fig. 5:**
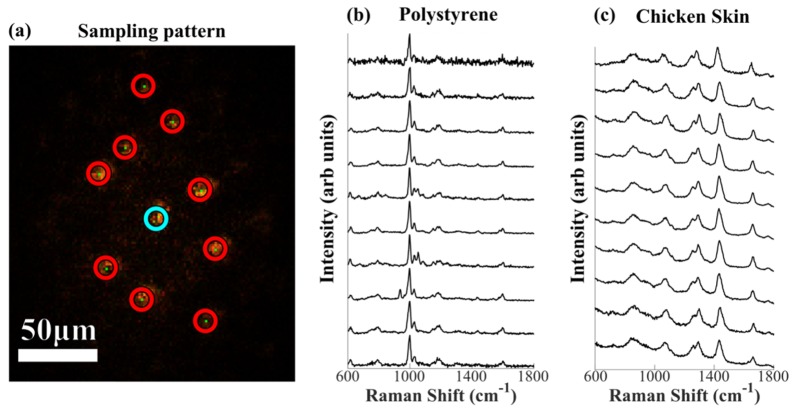
Multiplexed Raman spectra with an optimized blazing function based on 10 beams with total laser power ~1W at sample. Sampling positions selected manually. (a) Microscope camera image of sampling pattern (on polystyrene). (b) Uniform polystyrene film sample (acquisition time 0.1 s); (c) Chicken skin sample, acquisition time 2 seconds. Spectra plotted from binning two CCD tracks from raw image data with no additional processing. In (b) and (c) spectra are shifted vertically for clarity.

Chicken skin samples were used to investigate the ability of the multifocal Raman system to simultaneously measure spectra of animal tissue samples. Our previous work based on single-beam RMS, indicated that high diagnosis accuracies (sensitivity 95% and specificity 94%) for non-melanoma skin cancers was achieved when the Raman spectra had SNR >7 (signal: intensity of the 1450 cm^−1^ band, noise: r.m.s at ~1500 cm^−1^) [12]. For the single-beam RMS, the laser power is limited to ~120 mW, and the Raman spectra are typically acquired with an integration time of 2 seconds per spectrum [12]. For the multiplex RMS instrument described here, considering the total power of the Ti:Sapphire laser was 3.6W and the ~33% overall transmission efficiency of the optical components (from before the LC-SLM to the objective sample plane), the SLM was used to create at most 10 laser beams of ~100-120mW each (in reality the power distribution was not equal among beams as discussed above). For this reduced number of beams, the blazing function of the phase hologram displayed to the LC-SLM was optimized such that all beams excited the sample with a similar power. As the laser spot were ~1.5μm in diameter, the power density of each spot was estimated to 1.7 × 10^10^ Wm^−2^ (due to the microscopic separations of the laser spots, the power of each beam cannot be measured directly). Figure 5(b)

shows multiplexed Raman spectra of a polystyrene sample, showing that all 10 spectra had a more consistent SNR than those in Fig. 3. Figure 5(c) shows that under these optimized conditions, 10 multiplexed Raman spectra of a chicken skin sample can be measured within 2 seconds, and that the signal-to-noise ratio of all spectra was higher than 10 (signal: intensity of the 1450 cm^−1^ band, noise: r.m.s at ~1500 cm^−1^).

### 3.5 Multiplexed RMS and diagnosis of skin tissue samples excised during cancer surgery

A key feature of RMS is the ability to provide accurate and objective diagnosis of independent tissue samples based on multivariate spectral classifiers. To test whether the quality of the multiplexed Raman spectra was suitable for diagnosis, we built a simple classification model based on skin tissue samples from 10 patients undergoing skin cancer surgery, and then tested the performance of the classifier on multiplexed Raman spectra of skin samples from other patients. For the classification model, measurements were first carried out using a single laser beam (0th order beam) and raster scanning (similar to methods reported earlier in [12]).

Figure 6(a)

**Fig. 6:**
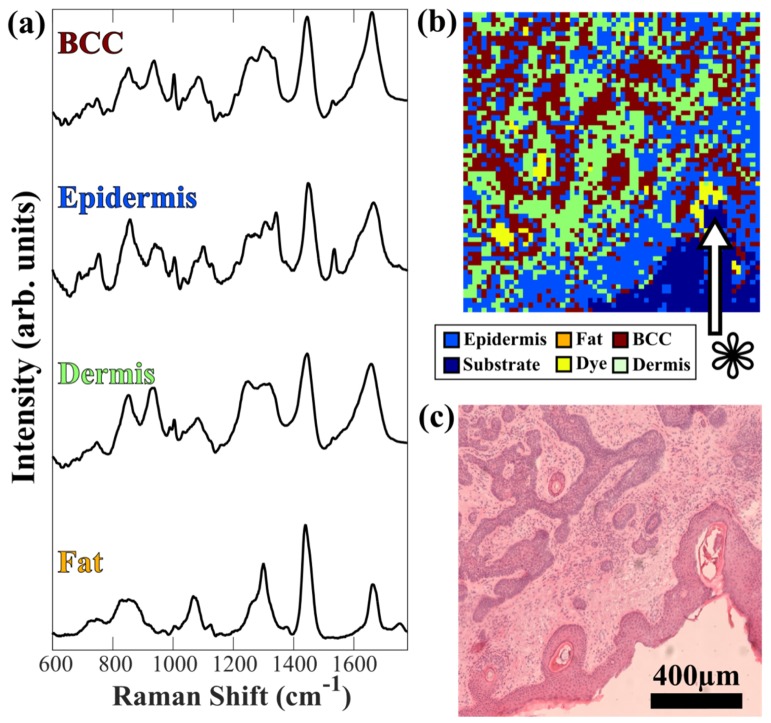
(a) Average Raman spectra of the healthy skin structures and BCC (samples from 10 patients, total number of Raman spectra was ~40,000). Spectra are shifted vertically for clarity. b). RMS diagnosis of skin surgical resection containing BCC using raster scanning and only the 0th order laser beam (15x15μm^2^ pixel size, false-positive on hair follicle marked by *) and (c) the adjacent H&E image.

presents the mean of the Raman spectra corresponding to the following classes used: basal cell carcinoma (BCC), epidermis, dermis and fat. In agreement with previous reports [4,12], the Raman spectra of BCC show more intense bands corresponding to DNA (e.g. O-P-O symmetric stretching 788 cm^−1^, PO2- stretching at 1098 cm^−1^) compared to other tissue structures, while the Raman spectra of dermis regions were dominated by bands specific to collagen fibers (e.g. 851 and 950 cm^−1^ bands assigned to proline and hydroxyproline) [26]. The Raman spectra of skin structures rich in lipids, such as sebaceous glands and fat regions, show specific bands characteristic to C-H, C-C and C = C vibrations (850 cm^−1^, 1070 cm^−1^, 1267 cm^−1^, 1301 cm^−1^, 1450 cm^−1^, ~1660 cm^−1^) [26]. Based on the spectral database, a classification model was developed based on peak-area multinomial linear regression. The performance of the model was evaluated by 5-fold cross-validation, indicating 94.9% sensitivity and 83.2% specificity for discrimination of BCC from healthy skin (all other classes combined).

Figure 6(b) shows a typical example of diagnosis image based on a single-beam (0th order beam) RMS raster scan and compares the result with the image of an adjacent tissue micro-section stained by H&E. This tissue was diagnosed with infiltrative BCC, and the classification was performed on each pixel, as being more representative to the accuracy of the diagnostic mode. Figure 6(b) shows that most tumor regions are correctly diagnosed, although some false positive pixels of BCC being diagnosed as epidermis and false positives (hair-follicle at the edge of epidermis, marked by *) can be observed. Such results are expected as the sensitivity and specificity were lower than 100%. Indeed, for a clinical application, the classification model would require Raman spectra from a larger number of patients and independent validation. Such extended model would capture better the inter-patient spectral variations and provide improved diagnosis. Nevertheless, the purpose here is to demonstrate the potential of obtaining objective and quantitative diagnosis based on the new multifocal Raman microscope.

Figure 7

**Fig. 7:**
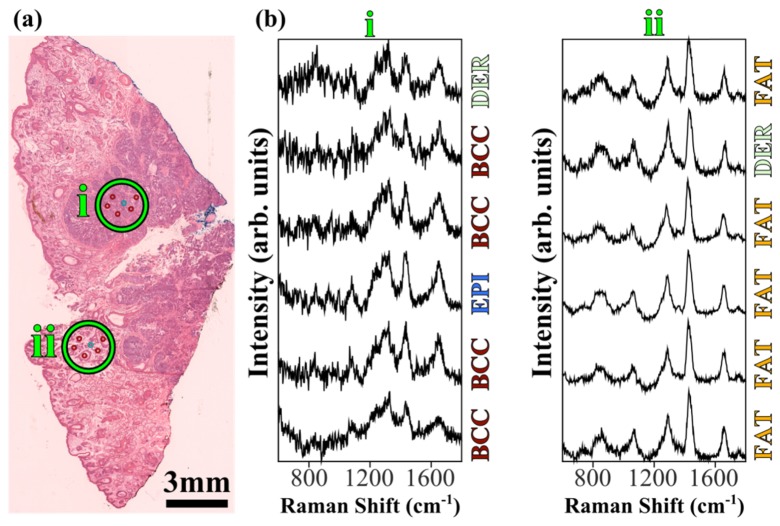
Example multiplex Raman measurements and diagnosis for a typical skin tissue sample from surgery. (a) Adjacent H&E tissue section, with the estimated measurement locations shown with green circles and manually selected sampling pattern (scaled up in size for clarity, not representative of exact sampling locations). (b) 6-beam multiplex Raman spectra (with baseline subtracted) from the labelled regions in the bright-field image, with corresponding diagnoses. Total laser power at sample: 1W; integration time: 2 seconds (batches of 6 sampling points). Spectra are shifted vertically for clarity.

presents typical multiplexed Raman spectra of a human skin tissue samples excised during Mohs micrographic surgery of BCC, and shows the diagnosis obtained by applying the classification model outlined in Fig. 6 to the individual multiplexed Raman spectra. In regions rich in fat, in agreement with Fig. 5(c) on chicken skin, Fig. 7(b) indicates that for an integration time of 2 seconds (similar to single-beam RMS), typically 6 Raman spectra can be acquired with a signal-to-noise ratio >7, and the diagnosis model had a high accuracy. However, in regions of skin where BCC was present, a strong fluorescence background was observed in the multiplexed Raman spectra, mainly caused by fluorescence emission by the dyes used for recording the orientation of the tissue required in Mohs surgery. Thus, the Raman spectra had a lower SNR and the diagnosis model provided an increased rate of false negatives. It is likely that this large background signal was more evident in the multiplexed Raman spectra because the 20 × /0.5NA objective used to increase the FOV of the multiplex RMS had a larger depth-of-field compared to the higher numerical aperture objectives used in previous studies based on single-beam RMS.

### 3.6 Automated multiplexed MSI of skin tissue excised during Mohs surgery

**Fig. 8:**
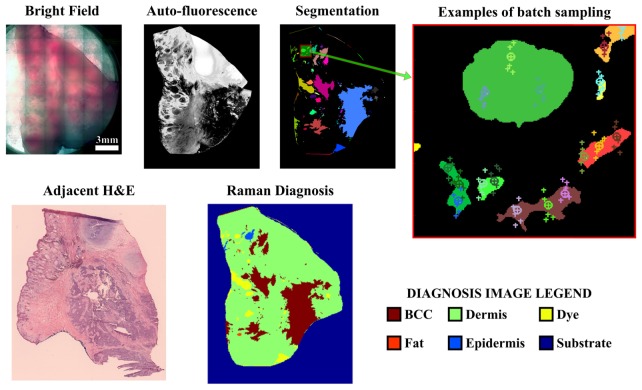
Automated multiplexed MSI diagnosis of skin tissue using segment-averaged spectra. The expanded view of the segmentation image shows typical batch sampling points with crosses, with the 0th order position marked with circles. Markers of the same color belong to the same batch. Each batch of six spectra was acquired in 2s.

To demonstrate the automated capability of the multiplexed system for MSI, skin resections obtained during Mohs micrographic surgery were first imaged using the confocal AF microscope and then transferred on the multiplexed RMS instrument. AF measurements were required in order to segment the tissue regions and generate sampling points for multiplexed RMS measurements. Figure 8(a)

shows that the confocal AF imaging has high sensitivity for detection of BCC (blue arrows) as tumours can be well delineated. However, the specificity of AF is low, as other tissue structures, such as epidermis, hair follicles, fat, sebaceous glands (green arrows), elicit similar AF intensity to BCC. Since the multiplex RMS was carried out on a separate instrument, the segmentation of the AF image was carried out after the coordinate transformation between the two microscopes was performed. This was based on stitched bright-field images of the tissue on the two microscopes, for which a transformation function was determined.

The same transformation was subsequently applied to the AF image, bringing it into the multiplex Raman instrument co-ordinate frame (with scaling between objective magnification taken into account). The segmentation routine was then performed on the transformed AF image, and sampling points for Raman were generated, with subsequent processing employed as described in section 2.1. For typical skin samples (size ~1cm^2^), the segmented AF image generated 600 – 900 sampling points. Additional points were generated in a batch at random locations to ensure 6 sampling locations were measured simultaneously, to ensure consistent power distribution for all batches. For the skin sample presented in Fig. 8, the segmented AF image generated 794 sampling points for RMS, resulting in 318 batches (1908 total sampling points for RMS) after processing. This corresponds to a total Raman measurement time of ~11 minutes for the whole tissue section (ignoring delays corresponding to microscope stage movement time, CCD readout, hologram calculation time and other software-based factors to the measurement duty-cycle; all these delays would typically double the acquisition time).

Figure 8 presents the diagnosis obtained by applying the classification model to multiplexed Raman spectra averaged across whole segments. Comparison of the diagnosis image obtained by multiplexed MSI and the adjacent H&E stained micro-section indicates that all BCC regions were correctly diagnosed. On the other hand, three false-positive regions (segments) were also identified in the image. Nevertheless, it is important to note that differences between adjacent tissue samples are expected also due to the 3-D structure of the tumours.

## 4. Conclusions

We have described a novel technique for true power-sharing multifocal RMS measurements from *ad hoc* software-generated sampling locations, obtaining spectra of sufficient quality to allow automated diagnosis of cancer margins in skin tissue. This was achieved by the coupling of a LC-SLM for laser-excitation and a DMD for Raman detection. The capability of the DMD as a multi-point reflective slit/pinhole was demonstrated for the Raman spectrometer, and shown to behave the same as a traditional mechanical slit. For a homogeneous polystyrene film (sample with high Raman scattering cross-section), 24 simultaneous Raman measurements were achievable with a total integration time of 1 second. For tissue samples with lower Raman scattering cross-section (chicken and human skin), an LC-SLM blazing function was used to evenly distribute the power between each sampling point, and was able to measure spectra with comparable SNR from 6 and 10 simultaneous points. The number of beams was lower than for polystyrene samples because higher laser power density is required for acquiring Raman spectra with suitable SNR. In principle, these numbers can be increased further with larger FOV optics and higher total laser power.

For human tissue specimens obtained in cancer surgery, the multifocal Raman modality was combined with auto-fluorescence imaging for selective-sampling Raman measurements. This allows several measurement options for decreasing the overall measurement time. We showed that it is possible to acquire multiple spectra simultaneously and obtain diagnosis on each individual spectrum, which speeds up the total measurement time by a factor proportional to the number of beams. Rather than restricting the laser to a single beam with power density limited by the photo-induced damage, the higher the laser power available, the larger the number of beams that can be generated, thus shorter the measurement times. We demonstrated the feasibility of obtaining diagnosis on large skin tissue samples typically obtained in surgery (1 cm2) in only 11 minutes, which is compatible with clinical use. Nevertheless, this approache would be particularly useful for faster automated cancer diagnosis on much larger tissue samples obtained during breast and head-and-neck cancer surgery, where typical tissue samples can be as larger as few centimeters.
